# Airborne Fine Particles and Risk of Hospital Admissions for Understudied Populations: Effects by Urbanicity and Short-Term Cumulative Exposures in 708 U.S. Counties

**DOI:** 10.1289/EHP257

**Published:** 2016-09-20

**Authors:** Mercedes A. Bravo, Keita Ebisu, Francesca Dominici, Yun Wang, Roger D. Peng, Michelle L. Bell

**Affiliations:** 1 School of Forestry and Environmental Studies, Yale University, New Haven, Connecticut, USA; 2 Biostatistics Department, Harvard University, Cambridge, Massachusetts, USA; 3 Johns Hopkins Bloomberg School of Public Health, Baltimore, Maryland, USA

## Abstract

**Background:**

Evidence of health risks associated with ambient airborne fine particles in nonurban populations is extremely limited.

**Objective:**

We estimated the risk of hospitalization associated with short-term exposures to particulate matter with an aerodynamic diameter < 2.5 μm (PM_2.5_) in urban and nonurban counties with population ≥ 50,000.

**Methods:**

We utilized a database of daily cardiovascular- and respiratory-related hospitalization rates constructed from Medicare National Claims History files (2002–2006), including 28 million Medicare beneficiaries in 708 counties. Daily PM_2.5_ exposures were estimated using the Community Multiscale Air Quality (CMAQ) downscaler. We used time-series analysis of hospitalization rates and PM_2.5_ to evaluate associations between PM_2.5_ levels and hospitalization risk in single-pollutant models.

**Results:**

We observed an association between cardiovascular hospitalizations and same-day PM_2.5_ with higher risk in urban counties: 0.35% [95% posterior interval (PI): –0.71%, 1.41%] and 0.98% (95% PI: 0.73%, 1.23%) increases in hospitalization risk per 10-μg/m^3^ increment in PM_2.5_ were observed in the least-urban and most-urban counties, respectively. The largest association for respiratory hospitalizations, a 2.57% (95% PI: 0.87%, 4.30%) increase per 10-μg/m^3^ increase in PM_2.5_, was observed in the least-urban counties; in the most-urban counties, a 1.13% (0.73%, 1.54%) increase was observed. Effect estimates for cardiovascular hospitalizations were highest for smaller lag times, whereas effect estimates for respiratory hospitalizations increased as more days of exposure were included.

**Conclusion:**

In nonurban counties with population ≥ 50,000, exposure to PM_2.5_ is associated with increased risk for respiratory hospitalizations; in urban counties, exposure is associated with increased risk of cardiovascular hospitalizations. Effect estimates based on a single day of exposure may underestimate true effects for respiratory hospitalizations.

**Citation:**

Bravo MA, Ebisu K, Dominici F, Wang Y, Peng RD, Bell ML. 2017. Airborne fine particles and risk of hospital admissions for understudied populations: effects by urbanicity and short-term cumulative exposures in 708 U.S. counties. Environ Health Perspect 125:594–601; http://dx.doi.org/10.1289/EHP257

## Introduction

Epidemiological studies have shown associations between fine particles (PM_2.5_) and health outcomes such as hospital admissions and mortality (Bell et al. 2008; Dominici et al. 2003, 2006; Kloog et al. 2014), but these studies are primarily based in urban areas owing to the placement of ambient monitors that provide the necessary exposure data. Consequently, the majority of PM_2.5_ health effects estimates are derived from urban populations. Although toxicological and individual- and population-level studies provide strong evidence that PM_2.5_ adversely affects health, key questions remain.

Current evidence on short-term PM_2.5_ exposure and health has critical limitations. First, estimates from many multi-city studies are obtained by pooling estimates from counties with monitoring data, thereby completely disregarding health effects in communities without monitoring data, which tend to be more rural (Bravo et al. 2012). This lack of monitoring data outside of urban areas precludes estimation of exposure and health effects in such locations, and as a result, it is unknown whether and to what extent health effects in monitored versus unmonitored, or urban versus rural, communities differ. Recently, researchers have used satellite data to estimate PM_2.5_ exposures (Kloog et al. 2011; Lee et al. 2016) and health outcomes in areas without monitoring data (Hyder et al. 2014; Kloog et al. 2014).

A second limitation is that by relying exclusively on monitoring data primarily from urban counties, studies cannot fully investigate susceptibility. Populations in urban counties differ demographically from those in nonurban (more rural) counties (Miranda et al. 2011) and may have dissimilar exposure levels or health responses to exposure. Regional and temporal differences have been observed in PM_2.5_ composition and health effect estimates (Bell et al. 2007); PM_2.5_ composition and time trends likely differ by urbanicity. Populations’ baseline health status and comorbidities (e.g., obesity), demographic and behavioral risk factors (e.g., tobacco use), and other factors differ between urban and nonurban communities. For example, rural communities have greater barriers to health care access (Vanasse et al. 2010), higher rates of many chronic diseases (Eberhardt and Pamuk 2004; Hartley 2004), and different activity patterns (Matz et al. 2015) than urban communities. Thus, PM_2.5_ exposures and susceptibility may differ between urban and nonurban populations, but such differences are not captured in currently available health effect estimates.

Third, most PM_2.5_ monitors sample every 3 days, prohibiting study of short-term cumulative exposures. Health effects of PM_2.5_ may depend on both the concentration and the duration of exposure. Same- or single-day lags of PM_2.5_ exposure may not fully capture health risk if the risk is affected by exposure experienced over multiple days, as some studies have suggested (Zanobetti et al. 2003).

Previous time-series PM_2.5_ studies, which are subject to the abovementioned limitations, studied up to 204 urban counties in the United States (Bell et al. 2008; Dominici et al. 2006). We utilized output from the Community Multi-scale Air Quality (CMAQ) model downscaler to estimate daily PM_2.5_ levels in monitored and nonmonitored areas for > 700 U.S. counties for 2002–2006. With daily downscaler-derived estimates of PM_2.5_, we estimated county-specific and overall health effects associated with short-term exposure to PM_2.5_ in populations excluded from previous studies. We also examined the health impacts of short-term cumulative exposures, which is only possible with daily PM_2.5_ estimates.

## Methods

### Health Data

We used files from the Centers for Medicare and Medicaid Services (CMS) to identify beneficiaries ≥ 65 years old who were enrolled in the Fee-for-Service plan for ≥ 1 month from 1 January 2002 to 31 December 2006. Using beneficiaries’ residential ZIP codes, we identified those who resided in 1 of the study area’s 795 U.S. counties with a population ≥ 50,000 in the 2000 U.S. Census (U.S. Census Bureau 2000a).

We linked this data set with CMS inpatient data to identify beneficiaries hospitalized with a principal discharge diagnosis of cardiovascular [*International Classification of Diseases, Ninth Revision, Clinical Modification* (ICD-9-CM) 390 to 459] or respiratory conditions (chronic obstructive pulmonary disease) (ICD-9-CM 490 to 492) or respiratory tract infections (ICD-9-CM 464 to 466, 480 to 487), from 1 January 2002 to 31 December 2006. Using dates of admission, we constructed our final sample of daily cardiovascular or respiratory hospital admission rates, aggregated at the county level (the data set identifying beneficiaries ≥ 65 years by county was used as the denominator in county-specific rate calculations). Of the 28,019,815 unique beneficiaries, 4,860,662 (17.3%) and 1,855,699 (6.62%) had at least one cardiovascular- or respiratory-related hospital admission, respectively, during the study period.

### Exposure Data

Daily (24 hr) averages of PM_2.5_ monitoring data (2002–2006) were obtained from the U.S. Environmental Protection Agency (EPA) National Air Monitoring Stations or State and Local Air Monitoring Stations (NAMS/SLAMS) network. Downscaler output was obtained for 2002–2006 (http://www.epa.gov/air-research/fused-air-quality-surfaces-using-downscaling-tool-predicting-daily-air-pollution). Inputs to the downscaler include monitoring data from the NAMS/SLAMS network and CMAQ numerical output, specifically, 24-hr PM_2.5_ concentrations at 12 km × 12 km grid cells simulated using CMAQ version 4.6 (Holland 2012). CMAQ is a sophisticated and extensively reviewed (Aiyyer et al. 2007; Amar et al. 2004, 2005) regional air quality model that estimates pollutant concentrations and deposition fluxes at local, regional, and continental scales. Using meteorological and emissions data, CMAQ simulates pollutant transformation, transport, and fate. Meteorological variables were estimated using 5th generation Penn State/NCAR Mesoscale Model version 3.6.3. The emissions inventory was based on the 2002 National Emissions Inventory and daily continuous emissions monitoring data for major point sources of nitrogen oxides (Holland 2012).

The downscaler uses monitoring data and gridded CMAQ output (12 km × 12 km) to estimate daily air pollution concentrations at census tract centroids using linear regression modeling with additive and multiplicative bias coefficients that can vary spatially and temporally (Berrocal et al. 2010a, 2010b, 2012). Downscaler estimates are used in the U.S. EPA’s Environmental Justice mapping and screening tool (EJSCREEN) (U.S. EPA 2015) and studies of air pollution and health (Gray et al. 2014). Although the downscaler was developed to provide predictive surfaces of air pollution for health studies relating daily pollution levels to daily health outcomes (Holland 2012), downscaler performance in locations without monitoring data, which correspond primarily to less-urban areas, is not well characterized. Thus, use of downscaler output allows us to estimate exposures and health effects in nonurban locations, but the resulting health effect estimates should be interpreted with care because there may be significant differences in downscaler performance in urban versus less-urban locations.

**Figure 1:**
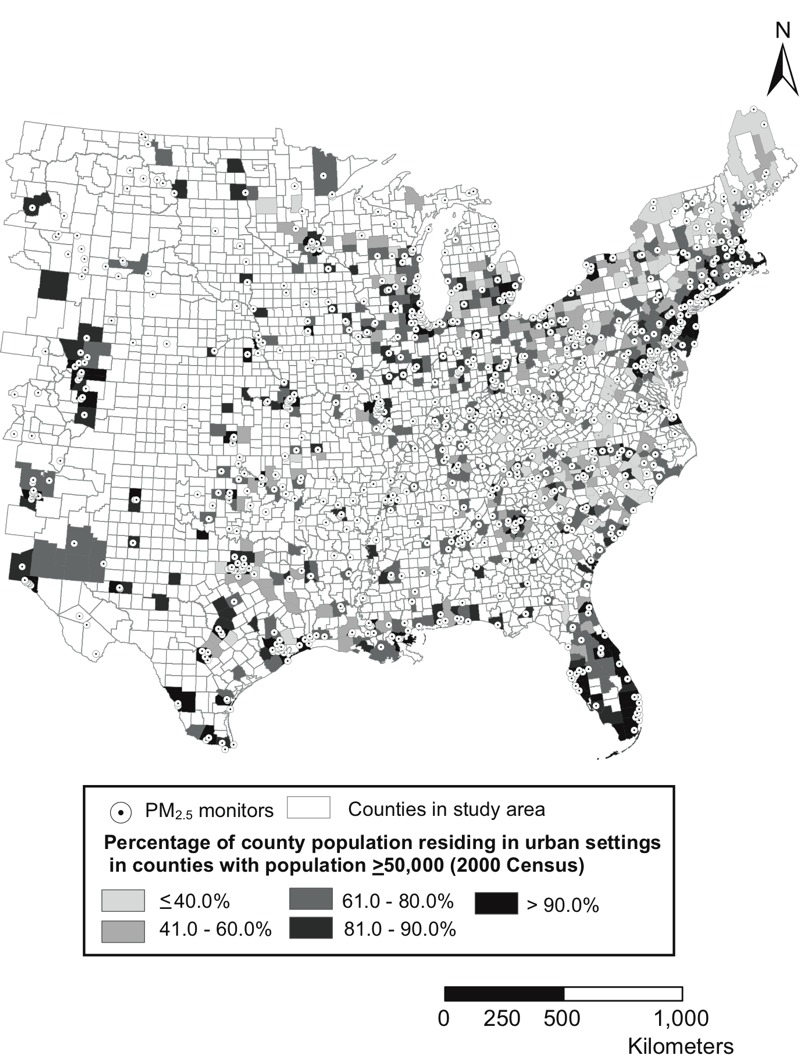
Percent of county population residing in urban areas. Urban populations reside in census blocks with (1) population density of ≥ 1,000 people/mi^2^ (386.1 people/km^2^) and (2) surrounding blocks with a density of ≥ 500 people/mi^2^ (193.1 people/km^2^). Rural populations are any population located outside of urban census blocks (U.S. Census Bureau 2000b). Shading indicates which counties were included in the study (*n *= 708 counties), with dark gray representing the most-urban counties and light gray representing the most-rural counties. Counties with the highest levels of urbanicity (> 90% of county population residing in urban settings) primarily correspond to counties containing or surrounding the following major cities: Houston, San Antonio, Austin, Odessa, Laredo, Brownsville, Corpus Christi, El Paso, and Dallas/Fort Worth, TX; Albuquerque, NM; Denver, Aurora, and Colorado Springs, CO; Omaha and Lincoln, NE; Tulsa and Oklahoma City, OK; Wichita and Kansas City, KS; Minneapolis–St. Paul, MN; Milwaukee, WI; Chicago, IL; St. Louis, MO; Fort Wayne and Indianapolis, IN; Detroit, MI; Buffalo and Schenectady, NY; Pittsburgh, PA; Nashville and Memphis, TN; Louisville and Lexington, KY; Cincinnati, Cleveland, Columbus, and Toledo, OH; Washington, DC; Norfolk, VA; Charlotte, Greensboro, and Raleigh, NC; Atlanta, GA; New Orleans and Baton Rouge, LA; and Tampa, Orlando, Miami, and Jacksonville, FL. There is a corridor of high-urbanicity counties along the eastern seaboard, extending roughly from Baltimore, MD to Boston, MA. The most-urban counties are often bordered, at least in part, by other counties with moderate to high levels of urbanicity (e.g., 41–90% of county population residing in urban settings). More-rural counties are more common in interior (i.e., non-coastal) areas of the southeast, including Oklahoma, the Northeast, the Ohio River Valley, and the Midwest. Ambient PM_2.5_ monitors are more likely to be sited in areas with higher levels of urbanicity. County boundaries are drawn according to Census 2000 Topologically Integrated Geographic Encoding and Referencing (TIGER)/Line files (https://www.census.gov/geo/maps-data/data/tiger-line.html). PM_2.5_, fine particulate matter.

We used downscaler output consisting of daily PM_2.5_ concentration estimates at census tracts for the eastern two-thirds of the United States, the region for which downscaler output are available for 2002–2006 (Figure 1). Further details on the downscaler methodology, results, and validation are available elsewhere (Berrocal et al. 2012).

We generated 24-hr county-level PM_2.5_ estimates using multiple approaches. We only estimated exposures for counties with populations ≥ 50,000 (*n* = 795) to ensure sufficient sample size. First, we used the standard approach of estimating exposures from monitoring data for counties with monitors (*n* = 446) and days with observations. Approximately 80% of PM_2.5_ monitors record observations once every 3 days. Multiple monitor measurements for the same day and county were averaged. Second, county-level 24-hr PM_2.5_ exposures were calculated from a population-weighted average of PM_2.5_ concentrations predicted by the downscaler at census tracts within each county using 2000 U.S. Census data (U.S. Census Bureau 2000a). These exposure estimates, hereafter referred to as “CMAQds,” were generated for 795 counties in the study area with a population ≥ 50,000 and all days in the study period (2002–2006). Lastly, we subset the CMAQds data set and calculated population-weighted county-level exposures *only* for counties and days with monitoring data. The data set of county-level PM_2.5_ exposures derived from downscaler output but restricted to days and counties with monitoring data is referred to as the “CMAQds_subset.”

Thus, we have three data sets of county-level exposure estimates derived from *a*) PM_2.5_ monitoring data, *b*) all available downscaler output (CMAQds), and *c*) downscaler output only in counties and on days with monitoring data (CMAQds_subset). The attributes of each PM_2.5_ data set and the methods used to estimate exposures are summarized in Table S1. We used metrics from the literature (Zhang et al. 2006) to assess whether monitor- and downscaler-derived exposure estimates were similar.

Counties were divided into five urbanicity categories based on percent of the county population residing in urban settings. According to the census, urban populations reside in census blocks with *a*) population density ≥ 1,000 people/mi^2^ (386.1 people/km^2^) and *b*) surrounding census blocks with population density ≥ 500 people/mi^2^ (193.1 people/km^2^); rural populations reside in blocks that do not meet these criteria (U.S. Census Bureau 2000b). Urban/rural categories are mutually exclusive, that is to say, 100 minus the percentage of the population residing in urban areas equals the percentage of the population residing in rural areas. The five categories of urbanicity consisted of counties with > 90%, 81–90%, 61–80%, 41–60%, and ≤ 40% of the population residing in urban settings. The percentage of the population in urban and rural (referred to here as “nonurban”) settings was obtained from the 2000 Census Summary File 3 (U.S. Census Bureau 2000a).

Daily temperature and dew point temperature data were obtained from the National Climatic Data Center (2012). Daily 24-hr estimates of temperature and dew point temperature for each county were generated from observations from all weather stations within the county. If a county did not have a weather monitor, weather data from the closest county within 30 mi (48.3 km) were used. Counties with insufficient meteorological data (*n* = 87) were removed from the analysis. This restriction resulted in 418 counties in the monitor and CMAQds_subset exposure data sets and 708 counties in the CMAQds exposure data set.

### Statistical Analysis

Health effects were estimated using two-stage Bayesian hierarchical modeling, an approach described elsewhere (Bell et al. 2004). In the first stage, log-linear Poisson regression models with over-dispersion were fit to county-specific time-series data on hospital admission rates and PM_2.5_ concentrations, adjusted for covariates. We chose covariates based on previous analyses (Dominici et al. 2006). Covariates included smooth functions (natural cubic spline) of same-day (day 0) temperature and dew point temperature [degrees of freedom (df) = 6], 3-day moving average of temperature and dew point temperature for days 1–3 (df = 3), and time to account for long-term trends in hospitalizations (df = 8/year), as well as categorical variables for age (65–74 years old, > 74 years old) and day of the week. The age variable was included to account for differential effects of air pollution by age, as has been done in previous studies (Bell et al. 2008). Lags for temperature and dew point temperature were consistent across all analyses.

In the second stage, we estimated the short-term association between PM_2.5_ and hospital admissions for the entire study area using two-level normal independent sampling estimation with noninformative priors (Everson and Morris 2000). This technique allowed us to combine relative risk estimates across counties while accounting for within-county statistical error and between-county variability in the true relative risks. The result was an overall effect estimate of the relationship between PM_2.5_ and hospital admissions across all counties. Alternatively, we could estimate the relationship between PM_2.5_ and hospitalizations for selected groups of counties that share characteristic(s) of interest, such as degree of urbanicity. Each hospitalization type (cardiovascular or respiratory) and PM_2.5_ data set (CMAQds, CMAQds_subset, or monitor-based estimates) was analyzed separately. County-level and overall (combined) effects were estimated for cardiovascular outcomes and respiratory outcomes at lag 0, lag 1 (previous day exposure), and lag 2. Effect estimates were compared to determine if they were significantly different based on the method of Schenker and Gentleman (2001).

To investigate whether PM_2.5_-hospitalization associations differed for single or multiple days of exposure, we fitted a distributed lag model with multiple lags of pollution (0- to 7-day lags) simultaneously included in the county-specific model. We then investigated whether effect estimates differed for more- versus less-urban counties using CMAQds-derived exposures, performing analyses stratified by the five urbanicity categories discussed previously.

The results are presented as the estimated percent increase in hospital admissions associated with a 10-μg/m^3^ increase in PM_2.5_ across a specified number of days. Statistical significance was assessed by the 95% posterior intervals (PI) excluding the value of zero. Statistical analyses were performed using R (version 3.2.1; R Project for Statistical Computing) and using the tlnise package for two-level normal independent sampling.

## Results

Observed concentrations are compared with CMAQds predictions in Table S2; see Figure S1 for a map of monitoring data availability by county. The mean daily county-level concentrations derived from the monitoring data and CMAQds_subset were 12.48 μg/m^3^ and 12.60 μg/m^3^, respectively. The mean and median within-county correlation between monitored and CMAQds_subset-predicted county-level concentrations were 0.96 and 0.97, respectively (standard deviation = 0.032; minimum and maximum = 0.72 and 0.99, respectively) (see Figure S2). The average normalized mean bias was < 1%, indicating that systematic bias in CMAQds_subset-predicted county-level PM_2.5_ concentrations was low.

PM_2.5_-hospitalization associations were estimated using exposures derived from *a*) monitoring data (*n* = 418 counties), *b*) CMAQds_subset (*n* = 418 counties), and *c*) CMAQds (*n* = 708 counties). County-specific maximum likelihood effect estimates resulting from the first-stage model using monitor- and CMAQds-derived exposure estimates are summarized in Figure S3. Overall estimates of cardiovascular and respiratory associations using different exposure data sets were similar (Table 1): based on CMAQds-derived exposure estimates, a 10-μg/m^3^ increase in PM_2.5_ was associated with a 1.16% (95% PI: 0.88%, 1.45%) increase in same-day (lag 0) respiratory admissions and a 0.79% (95% PI: 0.62%, 0.97%) increase in same-day cardiovascular admissions. Using CMAQds-derived exposure estimates, positive, statistically significant associations were observed for cardiovascular hospitalizations at lag 0 and for respiratory hospitalizations at lag 0, lag 1, and lag 2 (Table 1). Effect estimates from the different exposure data sets at the single lags examined (lag 0, 1, and 2) were similar in magnitude and were not significantly different from one another. For respiratory hospitalizations, lag 0 effect estimates tended to be larger than lag 1 or lag 2 effects regardless of the exposure estimates used. Cardiovascular effect estimates at lag 2 were negative when CMAQds_subset exposures were used [–0.20% (95% PI: –0.43%, –0.03%)].

**Table 1:** Percent increase in hospital admissions associated with a 10 μg/m^3^ increase in PM_2.5_ concentration, 2002–2006.

Health effect	Monitor data (*n *= 418 counties) Estimate (95% PI)	CMAQds_subset (*n *= 418 counties) Estimate (95% PI)	CMAQds (*n *= 708 counties) Estimate (95% PI)
Cardiovascular
Lag 0	0.87 (0.65, 1.09)*	0.98 (0.73, 1.23)*	0.79 (0.62, 0.97)*
Lag 1	0.15 (–0.06, 0.37)	0.15 (–0.09, 0.38)	–0.004 (–0.16, 0.15)
Lag 2	–0.14 (–0.36, 0.07)	–0.20 (–0.43, –0.03)*	0.09 (–0.06, 0.24)
Respiratory
Lag 0	1.10 (0.70, 1.50)*	1.11 (0.66, 1.56)*	1.16 (0.88, 1.45)*
Lag 1	0.37 (0.01, 0.78)*	0.38 (–0.02, 0.80)	0.29 (0.015, 0.58)*
Lag 2	0.57 (0.22, 0.93)*	0.57 (0.18, 0.96)*	0.37 (0.11, 0.63)*
Notes: CMAQds, Community Multi-Scale Air Quality downscaler exposure estimates; PI, posterior interval; PM_2.5_, fine particulate matter. *p < 0.05.			

**Figure 2:**
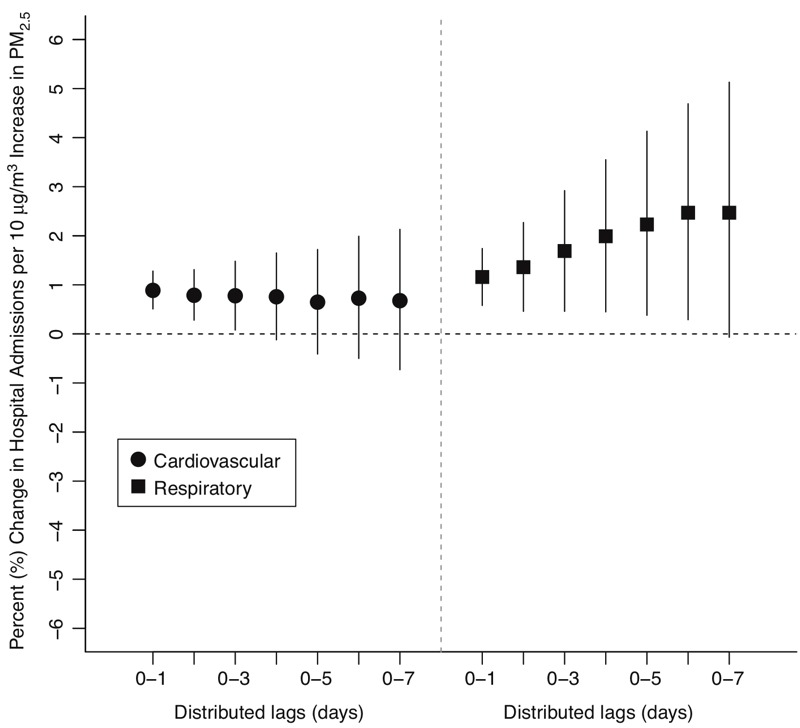
Percent increase in hospital admissions associated with a 10 μg/m^3^ increase in fine particulate matter (PM_2.5_) concentration, estimated for short-term distributed lags, using Community Multi-scale Air Quality (CMAQds) exposure estimates. Vertical lines represent 95% posterior intervals.

Daily CMAQds-derived exposure estimates allowed investigation of short-term cumulative lag effects. We used daily CMAQds-derived exposure estimates to include multiple single-day lags of PM_2.5_ concentration simultaneously in a distributed lag model, allowing pollution over multiple previous days to influence health (Peng and Dominci 2008). Health effects estimated for up to 7 days of multi-day lags (lag 01–07) using CMAQds-derived exposure estimates are presented in Figure 2. Point estimates for cardiovascular admissions decreased as more days were included in the lag structure but remained similar (range: 0.65–0.89% increase in admissions per 10 μg/m^3^ PM_2.5_ increase). Associations for respiratory hospitalizations were positive and statistically significant from lag 01 to lag 06, and effect estimates increased with additional days included in the lag through lag 07. The largest association was observed for lag 01 [0.89% (95% PI: 0.51%, 1.28%)] for cardiovascular admissions and lag 06 [2.47% (95% PI: 0.29%, 4.69%)] for respiratory admissions. Sensitivity analyses indicated that larger PM_2.5_ effects for respiratory outcomes at longer lag times were not attributed to uncontrolled temperature effects at longer lags. Lag results should be interpreted with caution: CMAQds-derived estimates may have greater day-to-day correlation than monitoring data because emissions inputs to CMAQ are correlated across time.

For the urbanicity analysis, we divided counties into five groups based on the percentage of county population residing in urban areas. Of 708 counties, 153 had > 90% of the population residing in urban settings (median population density = 477 people/km^2^), 113 counties had 81–90% of the population in urban areas (139 people/km^2^), 235 counties had 61–80% of the population in urban areas (78 people/km^2^), 140 counties had 41–60% of the population in urban areas (50 people/km^2^), and 67 counties had ≤ 40% of the population in urban areas (34 people/km^2^) (Figure 1). Mean PM_2.5_ for each of the urbanicity groups was not significantly different (Student’s *t*-test with Welch correction for unequal variances). Counties with > 90% or 61–80% of the population residing in urban areas had the highest average PM_2.5_ concentrations (12.5 μg/m^3^), and counties with ≤ 40% of the population residing in urban areas had the lowest concentration (11.8 μg/m^3^). The standard error of PM_2.5_ concentrations associated with downscaler predictions did not differ substantially by urbanicity (results not shown). Average (minimum–maximum) counts of daily county-level cardiovascular-related hospitalizations ranged from 1.88 (0–15) in the most nonurban counties to 13.8 (0–224) in the most urban counties. For respiratory hospitalizations, average (minimum–maximum) counts of daily county-level hospitalizations ranged from 0.73 (0–10) in the most nonurban counties to 4.44 (0–153) in the most urban counties.

**Figure 3:**
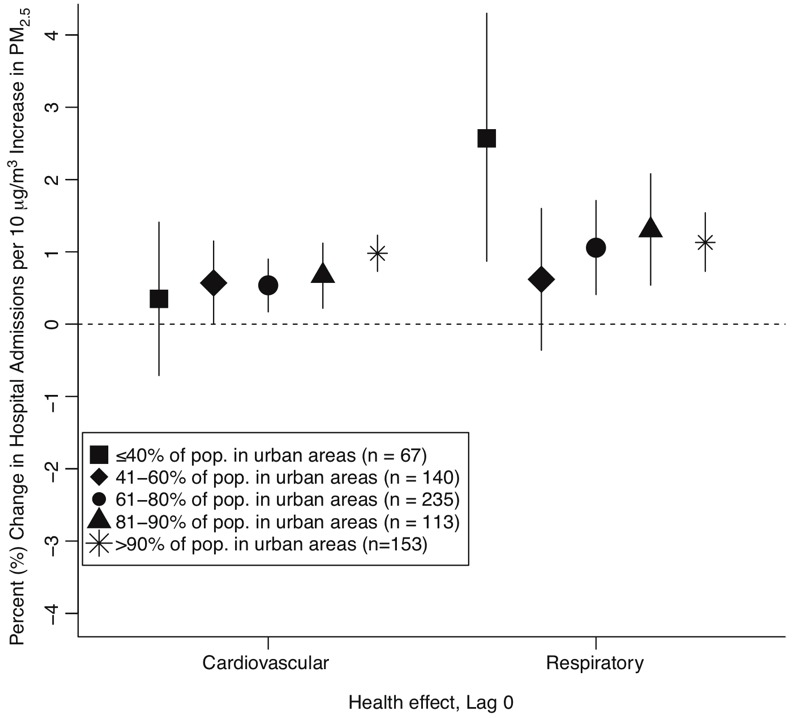
Percent increase in hospital admissions associated with a 10 μg/m^3^ increase in fine particulate matter (PM_2.5_) concentration, estimated for counties with different levels of urbanicity (lag 0). Vertical lines represent 95% posterior intervals.

Figure 3 shows health effect estimates by urbanicity category (lag 0), estimated using CMAQds exposure estimates. Cardiovascular effect estimates increased with increasing urbanicity. In contrast, the largest effect for respiratory hospitalizations [2.57% (95% PI: 0.87%, 4.30%) for a 10-μg/m^3^ increase in lag 0 PM_2.5_], was observed in counties with ≤ 40% of the population in urban areas. We also observed positive, statistically significant respiratory associations in some of the more-urban populations. Our findings indicate that cardiovascular effects were higher in the most-urban counties, whereas respiratory effects were highest in the least-urban counties. However, respiratory and cardiovascular effect estimates for counties with differing levels of urbanicity were not significantly different. We considered alternative groupings of urbanicity and found that categorizing urbanicity using four or five levels gave very similar results (results not shown). Health effect estimates by urbanicity estimated only for counties with monitoring data are provided in Figure S4.

## Discussion

Our principal findings include the following: *a*) evidence that PM_2.5_ may exert higher cardiovascular risk in urban populations; *b*) suggestive evidence that PM_2.5_ is more detrimental to respiratory health in nonurban populations; and *c*) evidence that respiratory health, more so than cardiovascular health, is affected by PM_2.5_ over the past few days. Our findings with respect to urban populations are consistent with those of previous studies focusing primarily on urban populations, which observed associations between short-term PM_2.5_ exposure and cardiorespiratory health (e.g., Dominici et al. 2006; Krall et al. 2013; Samet et al. 2000; Zanobetti et al. 2009). However, our findings also indicate that estimating risks using monitor data alone may underestimate the true effect across urban and nonurban populations, which occurs at a lag of a week (or longer) for respiratory hospitalizations.

Scientific evidence on urban and nonurban differences in PM_2.5_ composition is extremely limited (Kelly and Russell 2012), in part because of the dearth of monitors in less-urban areas. An analysis of hospitalizations and satellite-derived PM_2.5_ estimates in the mid-Atlantic United States found differences in associations between PM_2.5_ and cardiovascular hospitalizations in urban and rural populations (Kloog et al. 2014); others observed associations between respiratory health and urbanization (Ebisu et al. 2011). The urban–nonurban discrepancies in health response that we observed could have resulted from multiple factors, such as differences in exposure to pollutant mixtures (e.g., source-dependent PM_2.5_ composition), susceptibility to a given exposure in each population (e.g., baseline health status, access to or quality of health care, coexposures, comorbidities), and exposure measurement error.

Increasingly, evidence indicates that PM toxicity relates to chemical composition (Krall et al. 2013; Lippman et al. 2006) and source (Kelly and Russell 2012). PM_2.5_ chemical composition varies by geography, source, and season (Bell et al. 2007). Pollutant mixtures, and their associated toxicity, may differ by urbanicity (Schwab et al. 2004), which could affect observed associations. These variations could explain our findings that urban/nonurban differences in associations vary by cause of hospital admission because different chemical structures may affect health through different physiological pathways.

Urban and nonurban populations may have differential susceptibility to a given level of air pollution exposure (“effect modification”) (Greenland and Morgenstern 1989), which could relate to health care, lifestyle, activity patterns, and comorbidities or risk factors. Compared with urban areas, nonurban areas have higher poverty levels (Housing Assistance Council 2011), fewer physicians per capita, and greater transportation barriers to health care (Eberhardt et al. 2001; Vanasse et al. 2010). Distributions of comorbidities or risk factors in urban and nonurban populations may play a role in susceptibility to PM_2.5_. For example, a study of diabetes and coronary heart disease indicated that disease prevalence rates were higher in nonurban areas, but after adjusting for risk factors (e.g., poverty, obesity, tobacco use), prevalence was lower among respondents in nonurban areas than those in urban areas (O’Connor and Wellenius 2012). Lifestyle factors and activity patterns may also play a role: compared with nonurban residents, urban residents are more likely to engage in physical activity (Parks et al. 2003). Research in Canada found that rural populations spent significantly more time working outdoors (Matz et al. 2015). Such differences may affect not only susceptibility but also exposure levels.

Exposure measurement error may also contribute to differences in effect estimates for urban and nonurban counties. One key challenge is that evaluation of exposure estimates through comparison to monitoring data is limited in nonurban areas because of the lack of monitors. Validation of downscaler PM_2.5_ concentrations is only possible in locations with monitoring data; thus, it is not possible to evaluate downscaler performance in counties without ambient monitors, which tend to be less urban. However, less-urban areas are the very locations where exposure estimates are most needed. Zeger et al. (2000) identified three components of measurement error: *a*) difference between individual exposures and average personal exposure, *b*) difference between average personal exposure and ambient levels, and *c*) difference between measured and true ambient concentrations. The difference between downscaler-predicted and measured ambient concentrations is particularly relevant to our study. The downscaler incorporates information from ambient monitors, which are generally located in more urban settings, such that exposure estimates may have less measurement error in more-urban areas. One study of exposure measurement error in a time-series context such as ours indicated that larger differences between measured and true concentrations resulted in attenuated estimates of health risk (Goldman et al. 2011). However, depending on the error type (e.g., classical, Berkson), risk ratios could be attenuated or biased away from the null. Other issues (e.g., chemical composition, comorbidities) may be as important as or more important than measurement error.

Another principal finding is the lag structures observed for PM_2.5_ exposure and impacts on respiratory and cardiovascular hospitalizations. We found that the largest impact of PM_2.5_ on cardiovascular hospitalizations occurred at short lag time of 0–1 days, whereas the largest impact on respiratory hospitalizations occurred at a lag of a week (Figure 2). This finding is consistent with those of a previous study of particulare matter with an aerodynamic diameter ≤ 10 μm (PM_10_) (Zanobetti et al. 2003), in which the risk of respiratory mortality increased five-fold when PM_10_ exposure was characterized by longer distributed lags. Our findings with respect to lags are also consistent with those of several city-specific investigations that used daily air pollution data to evaluate lags between PM_2.5_ and cardiovascular- and respiratory-related morbidity and mortality, including studies in Denver, Colorado; Seattle, Washington (Kim et al. 2012); and Detroit, Michigan (Zhou et al. 2011), among others (Schwartz 2000). This is a critical point because it is not possible to estimate the health impacts of short-term cumulative exposures in most U.S. locations using traditional methods given that very few monitors measure PM_2.5_ daily. Of 708 counties in the present analysis, only 57 (8.1%) had > 90% of days with monitoring data. As a result, any analysis of cumulative exposures using monitoring data is necessarily constrained to areas with daily monitoring data, which are overwhelmingly urban.

Moreover, our analysis indicated that counties for which short-term cumulative exposure and health effects could be estimated using monitor-derived exposures (i.e., primarily urban counties with daily data), had lower effect estimates for respiratory hospitalizations than other counties (i.e., those with less PM_2.5_ monitoring data availability) (results not shown). Thus, respiratory health effects modeled using distributed lag exposures obtained from counties with near-complete monitoring data may not be generalizable to counties with few or no monitoring data and may in fact underestimate health effects in such counties.

Our study has several limitations. Our analysis was restricted to counties with populations ≥ 50,000 in the 2000 U.S. Census, which limits how nonurban included counties can be because more sparsely populated rural counties often have populations < 50,000. This analytical design was chosen for sample size considerations, and our findings indicate that further investigation of health impacts of air pollution in nonurban populations is warranted. Although we evaluated CMAQds performance with respect to monitoring data, CMAQds performance cannot be evaluated in areas with limited or no monitoring data. Differences between CMAQds-derived exposure estimates and monitor-derived exposure estimates may be greater in less-urban counties because there are few or no monitoring data to use as input to the downscaler in less-urban areas. Clearly, there is less opportunity to validate CMAQds-derived exposure estimates in places without monitors, the very places where exposure estimates are most needed. The potential difference in error between urban and less-urban counties means that differences in risk estimates for urban and less-urban counties must be interpreted with caution, and this is an area in which the need for further research is acute. We do not have substantial data on spatial variability or urban/nonurban differences in PM_2.5_ composition or copollutant concentrations or mixtures. Additionally, although the ≥ 65 years of age demographic is one of the fastest-growing segments of the U.S. population (Ortman et al. 2014), and older individuals may have heightened susceptibility to air pollution (Sacks et al. 2011), health effects estimated for elderly individuals are not necessarily generalizable to the U.S. population or to other potentially susceptible populations. This study was limited to areas were CMAQds-predicted concentrations were available for 2002–2006.

Strengths of this study include the investigation of several important questions that were not addressed in previous studies: for example, health effect estimates in nonurban counties were not addressed because monitors tend to be sited in urban locations; furthermore, health effects of short-term cumulative exposures (distributed lags) were not investigated. A major strength of this study is inclusion of understudied populations residing in nonurban areas: previous multi-city studies of air pollution have been based exclusively in more-urban counties and communities, and city-specific studies also tend to focus on metropolitan areas (Zanobetti and Schwartz 2005). Another significant strength is our analysis of short-term distributed lags in areas with nondaily monitoring data or with no data. Future work could incorporate uncertainty associated with downscaler predictions into the exposure and health effect estimates; investigate whether results are affected by comorbidities, lifestyle, or risk factors that may affect susceptibility to air pollution or cardiovascular or respiratory disease; and examine even more rural counties by pooling populations in adjacent counties or by similar methods.

## Conclusion

As a result of urban bias in epidemiological studies, which is largely driven by data availability, the health impacts of air pollution in nonurban locations are largely unknown. Health effect estimates for predominantly urban populations may not be generalizable to nonurban areas, where > 59 million people live in the United States. Further, estimating health effects of PM_2.5_ with nondaily data may underestimate true health effects, particularly for respiratory-related hospitalizations. Health analyses in urban locations, with large populations and high pollution levels, are useful from public health impact and regulatory perspectives; however, health outcomes for large numbers of people remain poorly understood. Our findings indicate significant respiratory health impacts in nonurban areas and over a multiday exposure period. Additional research is needed to investigate the health impacts of air pollution on nonurban populations and to explore the differences in health effect estimates presented here.

**Editor’s Note:** In the Advance Publication, the images were reversed between Figures 2 and 3. The images and captions are now in the correct positions.

## Supplemental Material

(1.9 MB) PDF
