# Supplementary material for: Airborne Fine Particles and Risk of Hospital Admissions for Understudied Populations: Effects by Urbanicity and Short-Term Cumulative Exposures in 708 U.S. Counties
Source: Environ Health Perspect. 2016 Sep 20;125(4):594–601. doi: 10.1289/EHP257 (PMC5381978; doi:10.1289/EHP257)
Supplement: (1.9 MB) PDF [file EHP257.s001.acco.pdf]

Environ Health Perspect
DOI: 10.1289/EHP257

Note to readers with disabilities: *EHP* strives to ensure that all journal content is accessible to all readers. However, some figures and Supplemental Material published in *EHP* articles may not conform to 508 standards due to the complexity of the information being presented. If you need assistance accessing journal content, please contact ehp508@niehs.nih.gov. Our staff will work with you to assess and meet your accessibility needs within 3 working days.

Supplemental Material

Airborne Fine Particles and Risk of Hospital Admissions for Understudied Populations: Effects by Urbanicity and Short-Term Cumulative Exposures in 708 U.S. Counties

Mercedes A. Bravo, Keita Ebisu, Francesca Dominici, Yun Wang, Roger D. Peng, and Michelle L. Bell

Table of Contents

Table S1. Characteristics of $PM_{2.5}$ pollution data.

Table S2. Summary statistics of model evaluation for 24-h average $PM_{2.5}$ county level exposure estimates

Figure S1. Availability of monitor data by county for the study area output (data from 2000 U.S. Census; map created using ArcGIS).

Figure S2. County-specific correlation between exposure estimates derived from observed data and CMAQds simulated $PM_{2.5}$ concentrations for the 418 counties with monitoring data and populations ≥50,000, 2002-2006 (data from 2000 U.S. Census; map created using ArcGIS). This map shows correlations between county level monitor-derived daily exposure estimates for daily county level CMAQds-derived exposure estimates. Only counties with monitoring data and populations ≥50,000 are included in the comparison

Figure S3. Comparison of county-specific maximum likelihood health effect estimates obtained from monitor-derived exposure estimates (x-axis) versus CMAQds_subset-derived exposure estimates ($\hat{\beta}^c$coefficients relating $PM_{2.5}$ concentration to hospitalization rates in county-specific regression model): (a) cardiovascular; (b) respiratory (n=418 counties) Point size is inversely proportional to the magnitude of the standard error associated with each monitor-derived county-specific maximum likelihood health effect estimate, such that a smaller point size indicates greater uncertainty associated with that county-specific estimate. Note that these values represent coefficients that have not been scaled.

Figure S4. Percent increase in hospital admissions associated with a 10μg/m^3 increase in $PM_{2.5}$ concentration, estimated using monitoring data (gray) and downscaler output (black), only for counties with monitoring data (CMAQds_subset), by level of urbanicity (lag 0) Vertical lines represent 95% posterior intervals. Urbanicity is measured as percent of county population residing in nonurban areas

Tables

Table S1. Characteristics of $PM_{2.5}$ pollution data/output

	Monitoring data	CMAQds output
**Data description**	Federal Reference Method ambient air quality monitors	Daily predictions of pollutant concentrations at Census Tracts centroids from combination of ambient monitoring data and CMAQ v4.6 output
**Spatial form of concentration observation/estimate**	Point	Point
**Spatial resolution of original dataset**	Variable	Variable
**Temporal resolution**	Variable, ~1 observation/3 days	Daily, every day
**Method(s) used to estimate county level concentration**	Monitor(s) within given county averaged	Population weighted Census Tracts to estimate county level conc.
**Spatial coverage of exposure estimates**	~418 counties	~2,818 counties

Table S2. Summary statistics of model evaluation for 24-h average $PM_{2.5}$ county level exposure estimates[a,b]

EvaluationMetric	Value
Mean daily county level concentration	
CMAQds	12.28 $\mu g/m^3$
CMAQds_subset	12.60 $\mu g/m^3$
Observed (monitor-derived)	12.48 $\mu g/m^3$
Normalized mean bias (NMB) (%)	0.95%
Normalized mean error (NME) (%)	9.75%
Mean correlation (standard deviation)	0.97 (0.032)

[a] Formulas and further description of metrics of model performance are presented in Zhang et al. 2006.
[b] The mean correlation refers to the mean correlation between monitor-derived and CMAQds-derived exposure estimates within a county (and not correlations across all counties and days).

Figures

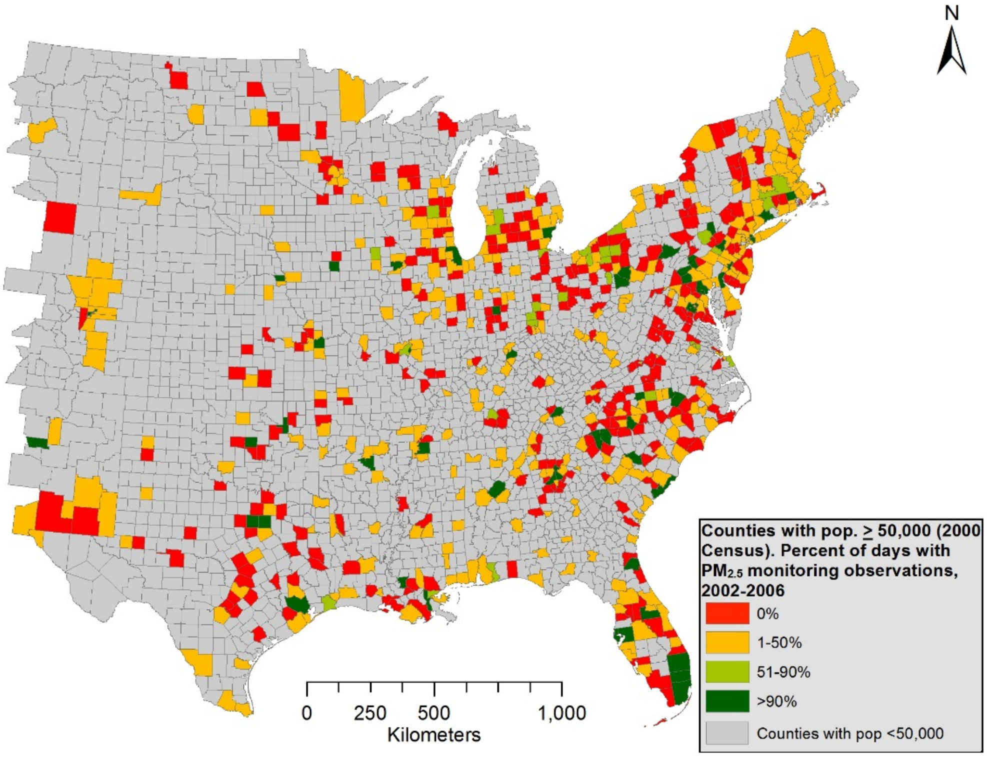

Figure S1. **Availability of monitor data by county for the study area (data from 2000 U.S. Census; map created using ArcGIS).**

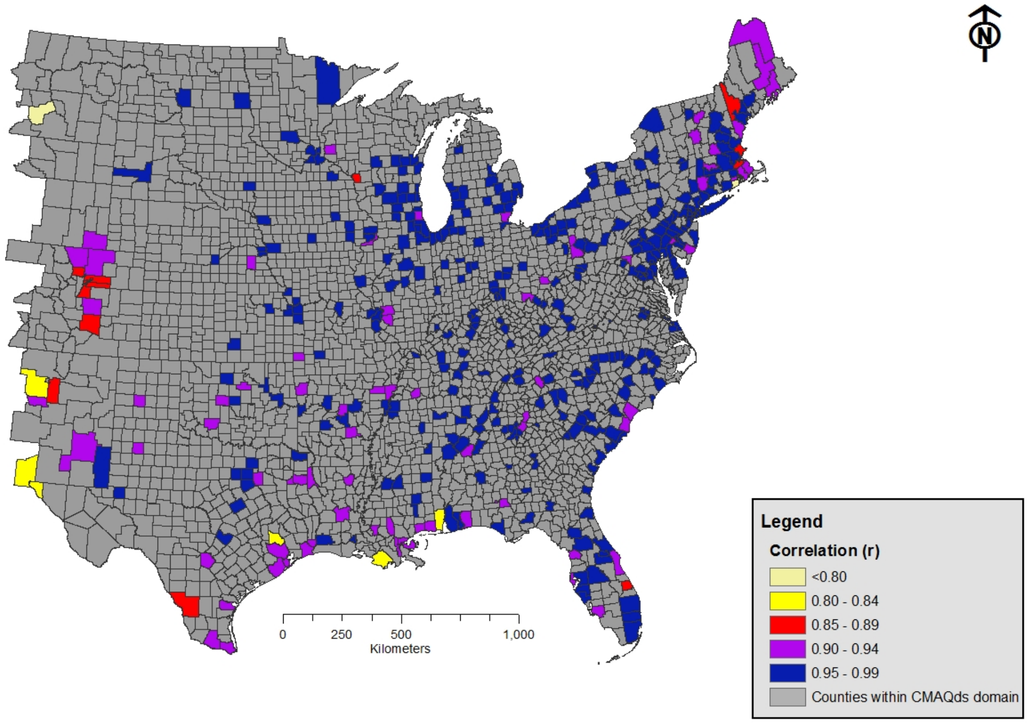

Figure S2. County-specific correlation between exposure estimates derived from observed data and CMAQds simulated $PM_{2.5}$ concentrations for the 418 counties with monitoring data and populations ≥50,000, 2002-2006 (data from 2000 U.S. Census; map drawn using ArcGIS). This map shows correlations between county level monitor-derived daily exposure estimates for daily county level CMAQds-derived exposure estimates. Only counties with monitoring data and populations ≥50,000 are included in the comparison

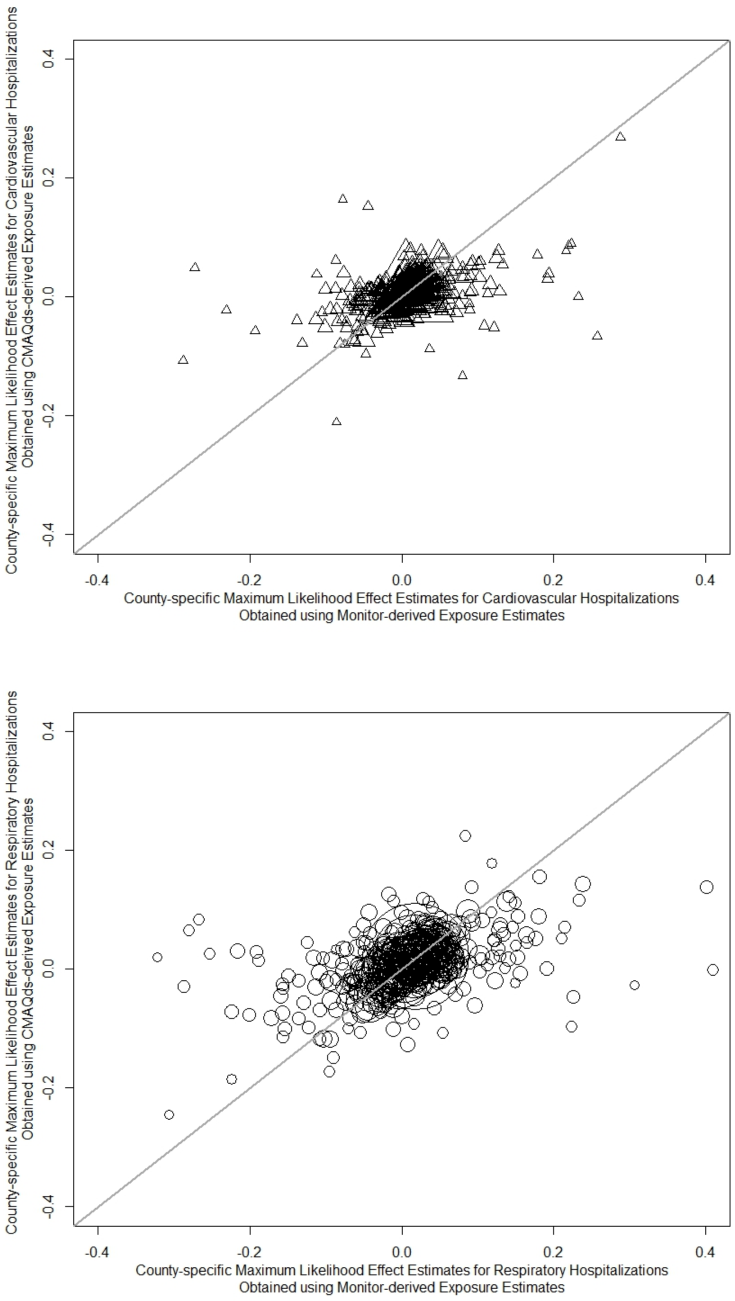

Figure S3. Comparison of county-specific maximum likelihood health effect estimates obtained from monitor-derived exposure estimates (x-axis) versus CMAQds_subset-derived exposure estimates ($\widehat{\boldsymbol{\beta}}^{c}$coefficients relating $PM_{2.5}$ concentration to hospitalization rates in county-specific regression model): (a) cardiovascular; (b) respiratory (n=418 counties). Point size is inversely proportional to the magnitude of the standard error associated with each monitor-derived county-specific maximum likelihood health effect estimate, such that a smaller point size indicates greater uncertainty associated with that county-specific estimate. Note that these values represent coefficients that have not been scaled.

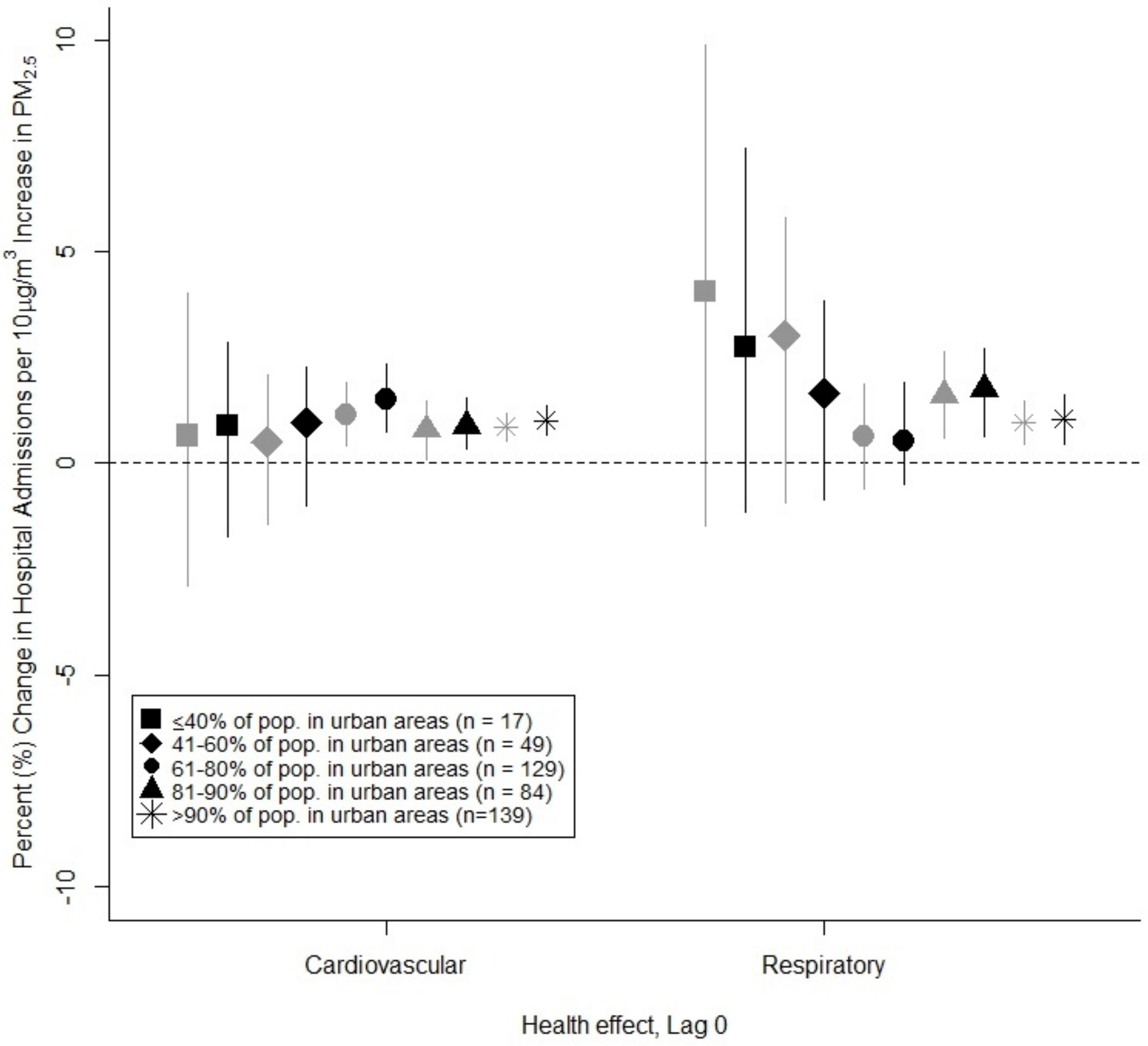

Figure S4. Percent increase in hospital admissions associated with a 10µg/m^3 increase in PM$_{2.5}$ concentration, estimated using monitoring data (gray) and downscaler output (black), only for counties with monitoring data (CMAQds_subset), by level of urbanicity (lag 0). Vertical lines represent 95% posterior intervals. Urbanicity is measured as percent of county population residing in nonurban areas.

References

Zhang Y, Liu P, Pun B, Seigneur C. 2006. A comprehensive performance evaluation of MM5-CMAQ for the Summer 1999 Southern Oxidants Study episode - Part I: Evaluation protocols, databases, and meteorological predictions. Atmos Environ 40: 4825-4838.
